# Tetra­sodium (dihydrogenhepta­oxido­digermanato)bis­(dihydrogentetra­oxido­germanato)dicopper(II) monohydrate

**DOI:** 10.1107/S1600536809007715

**Published:** 2009-03-11

**Authors:** Ya-Feng Li, Dan-Ping Li, Cui-Li Shi, Yong-Sheng Hu, Li Jin

**Affiliations:** a School of Chemical Engineering, Changchun University of Technology, Changchun 130012, People’s Republic of China

## Abstract

In the hydro/solvothermally synthesized title compound, Na_4_[Cu_2_(H_2_Ge_2_O_7_)(H_2_GeO_4_)_2_]·H_2_O, the framework building units include CuO_4_, GeO_2_(OH)_2_ and GeO_3_(OH) tetra­hedra, the latter being condensed into H_2_Ge_2_O_7_^4−^ dimers. All the tetra­hedra are connected by corner-sharing into four-membered-ring (4MR) secondary building units containing two CuO_4_, one GeO_2_(OH)_2_ and one GeO_3_(OH) entity. The 4MRs form chains by corner-sharing the Cu unit and adjacent chains are linked by H_2_Ge_2_O_7_^4−^ dimers, generating layers containing ten-membered rings. Three sodium cations (one with site symmetry 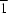 and one with site symmetry 2) and a water mol­ecule (O-atom site symmetry 2) complete the structure. A network of O—H⋯O hydrogen bonds helps to consolidate the packing.

## Related literature

For related structures, see: Bu *et al.* (2000 ▶); Cascales *et al.* (1999 ▶); Hartman & Kevan (1999 ▶); Julius *et al.* (2003 ▶); Li *et al.* (2000 ▶); O’Keeffe & Yaghi (1999 ▶); Whitfield *et al.* (2003 ▶).

## Experimental

### 

#### Crystal data

Na_4_[Cu_2_(H_2_Ge_2_O_7_)(H_2_GeO_4_)_2_]·H_2_O
*M* *_r_* = 773.56
Orthorhombic, 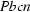

*a* = 13.041 (3) Å
*b* = 8.7440 (17) Å
*c* = 12.975 (3) Å
*V* = 1479.6 (6) Å^3^
*Z* = 4
Mo *K*α radiation
μ = 11.05 mm^−1^
*T* = 293 K
0.20 × 0.15 × 0.05 mm

#### Data collection

Stoe IPDS diffractometer
Absorption correction: numerical (*X-RED*; Stoe & Cie, 1997 ▶) *T*_min_ = 0.152, *T*_max_ = 0.582
9596 measured reflections
1441 independent reflections
1038 reflections with *I* > 2σ(*I*)
*R*_int_ = 0.099

#### Refinement

*R*[*F* ^2^ > 2σ(*F* ^2^)] = 0.041
*wR*(*F* ^2^) = 0.118
*S* = 1.03
1441 reflections
129 parameters
1 restraint
H atoms treated by a mixture of independent and constrained refinement
Δρ_max_ = 2.11 e Å^−3^
Δρ_min_ = −1.17 e Å^−3^

### 

Data collection: *EXPOSURE* in *IPDS Software* (Stoe & Cie, 1997 ▶); cell refinement: *CELL* in *IPDS Software*; data reduction: *INTEGRATE* in *IPDS Software*; program(s) used to solve structure: *SHELXS97* (Sheldrick, 2008 ▶); program(s) used to refine structure: *SHELXL97* (Sheldrick, 2008 ▶); molecular graphics: *DIAMOND* (Brandenburg, 2000 ▶); software used to prepare material for publication: *SHELXL97*.

## Supplementary Material

Crystal structure: contains datablocks I, global. DOI: 10.1107/S1600536809007715/hb2913sup1.cif

Structure factors: contains datablocks I. DOI: 10.1107/S1600536809007715/hb2913Isup2.hkl

Additional supplementary materials:  crystallographic information; 3D view; checkCIF report

## Figures and Tables

**Table 1:** Selected bond lengths (Å)

Ge1—O1	1.721 (4)
Ge1—O4	1.724 (4)
Ge1—O3	1.781 (4)
Ge1—O2	1.795 (4)
Ge2—O7^i^	1.724 (4)
Ge2—O8	1.729 (3)
Ge2—O6	1.772 (2)
Ge2—O5	1.794 (4)
Cu1—O4	1.953 (4)
Cu1—O1^ii^	1.960 (4)
Cu1—O8	1.961 (4)
Cu1—O7	1.964 (4)

Symmetry codes: (i) 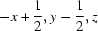; (ii) 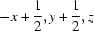.

**Table 2:** Hydrogen-bond geometry (Å, °)

*D*—H⋯*A*	*D*—H	H⋯*A*	*D*⋯*A*	*D*—H⋯*A*
O2—H2⋯O6^iii^	0.82	2.59	3.298 (5)	146
O3—H3⋯O1^iv^	0.82	1.98	2.765 (6)	161
O5—H5⋯O4^v^	0.82	1.94	2.755 (6)	171
O1*W*—H1⋯O7	0.85 (4)	1.96 (5)	2.773 (6)	159 (6)

Symmetry codes: (iii) 
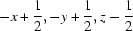
; (iv) 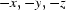; (v) 
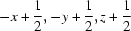
.

**Fig. 1.**
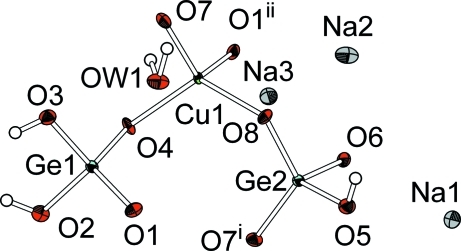
A fragment of (I), showing displacement ellipsoids for the non-hydrogen atoms at the 50% probability level. [Symmetry codes: (i) 0.5 - x, -1/2 + y, z; (ii) 0.5 - x, 1/2 + y, z.]

**Fig. 2.**
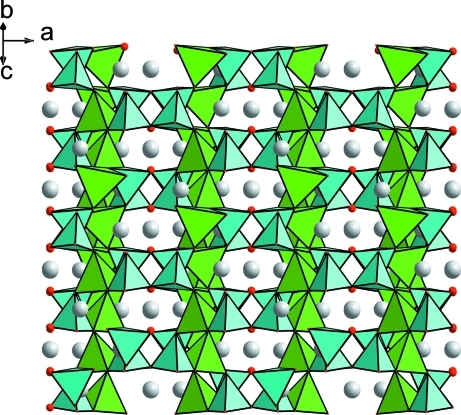
The packing diagram of (I), viewed along [021] direction. CuO4 tetrahedra are shown in green, GeO4 tetrahedra in cyan, Na atoms in light gray and O atoms in red. H atoms are omitted for clarity.

## supplementary crystallographic information

### Comment

Recently, gernamates have been recieving the extensive attentions owing to their potential applications such as ion-exchange, catalyst and sorption. The four-coordinated Ge has a trendency to form the double four ring geometry with oxygen, which can be observed in several Ge-based zeolites such as ASV, BEC, IWR, IWW, UOZ (O'Keeffe, *et al.*, 1999). This derives from flexible Ge—O—Ge bond angles (130° or so). Incorporation of transition metals into Si-based materials is extremely interesting because transition metals can improve zeolite properties (Hartman, *et al.*, 1999). Following the successful introduction of transition metals into zeolite materials, efforts are also made to incorporate transition metal elements, such as V (Whitfield, *et al.*, 2003), Co (Julius, *et al.*, 2003), Cu (Cascales, *et al.*, 1999), Zn (Bu, *et al.*, 2000) and Zr (Li, *et al.*, 2000), into germanate frameworks. In this paper, a new copper(II) germanate with 10MR network, Na_4_[Cu_2_Ge_4_O_9_(OH)_6_].H_2_O (I), is described.

The asymmetric unit of (I) comprises two crystallographically independent GeO_4_–GeO_2_(OH)_2_ and GeO_3_(OH), one CuO_4_, three Na cations one of which locates on the center of *ac* plane, and a half of free water molecule (Fig.1). All of Ge and Cu atoms are tetrahedrally coordinated by oxygen atoms. Both GeO_4_ and CuO_4_ tetrahedra are lightly distorted with 1.721–1.794 Å of Ge—O and 1.952–1.964 Å of Cu—O. There is no linkage between Ge1 and Ge2, but Ge2 can really form the dimer of H_2_Ge_2_O_7_^4-^ by sharing O6 with 124.89° of Ge—O—Ge.

The 4MR SBU of (I) is consisted of two CuO_4_, one GeO_2_(OH)_2_ and one GeO_3_(OH). The GeO_4_ tetrahedra and CuO_4_ tetrahedra are connected by sharing corner. The bond angles of Ge—O—Cu range from 117.72° to 122.65°. The corner-sharing '4-rings' chain is constructed by the CuO_4_ of 4MR SBU as the corner. In such a chain, every CuO_4_ has four Cu—O—Ge linkages, while either GeO_2_(OH)_2_ or GeO_3_(OH) just have two Cu—O—Ge linkages. As the result of the existence of H_2_Ge_2_O_7_^4-^, the adjacent corner-sharing '4-rings' chains are connected to the layer of 10MR net (Fig.2). Three crystallographically independent Na atoms, one of which loacates on the special position (1/2,0,1/2), act as the balanced cation and interact with the framework by Na—O electrostatic interactions with 2.372–2.569 Å of Na—O. Except for intramolecular hydrogen bond (O2—H2···O6^i^, O3—H3···O1^ii^, O5—H5···O4^iii^), there exists the intermolecar hydrogen bond between water molecules and terminal hydroxide anions (O1W—H1···O7).

### Experimental

GeO_2_(0.25 g), Cu(Ac)_2_.3H_2_O (0.28 g) and NaOH (0.38 g) were successively added into a pyridine/water (7.5 ml/1 ml) solution with molar ratio of 1 GeO_2_:0.5 Cu(Ac)_2_.3H_2_O:4 NaOH: 23 H_2_O: 38 pyridine. The deep blue mixture was vigoursly stirred for 6 hr. The final mixture was sealed into 23 ml autoclave and heated up to 438 K for 6 days. The autoclave was naturally cooled to room temperature, and the product was filtered, washed by distilled water and alcohol and dried at room temperature. Deep blue prismatic large single crystals of (I) were obtained. The atomic ratio of Ge: Cu: Na determined by EDX was 2:1:2, in agreement with the results of structural determination of (I).

### Refinement

The H atoms of –OH groups were placed in ideal positions and refined as riding atoms with O—H = 0.82 Å. The water H atom was located in a difference map and refined with restraints of O—H = 0.85 (1) Å and H···H = 1.37 (2) Å.

### Crystal data

**Table:**

Na_4_[Cu_2_(H_2_Ge_2_O_7_)(H_2_GeO_4_)_2_]·H_2_O	*F*(000) = 1464
*M**_r_* = 773.56	*D*_x_ = 3.472 Mg m^−^^3^
Orthorhombic, *P**b**c**n*	Mo *K*α radiation, λ = 0.71073 Å
Hall symbol: -P 2n 2ab	Cell parameters from 2000 reflections
*a* = 13.041 (3) Å	θ = 2.8–26.0°
*b* = 8.7440 (17) Å	µ = 11.05 mm^−^^1^
*c* = 12.975 (3) Å	*T* = 293 K
*V* = 1479.6 (6) Å^3^	Block, dark blue
*Z* = 4	0.20 × 0.15 × 0.05 mm

### Data collection

**Table:**

Stoe IPDS diffractometer	1441 independent reflections
Radiation source: fine-focus sealed tube	1038 reflections with *I* > 2σ(*I*)
graphite	*R*_int_ = 0.099
Detector resolution: 6.0 pixels mm^-1^	θ_max_ = 26.0°, θ_min_ = 2.8°
φ–oscillation, φ–incr.=1.8°, 100 exposure scans	*h* = −16→15
Absorption correction: numerical (*X-RED*; Stoe & Cie, 1997)	*k* = −10→10
*T*_min_ = 0.152, *T*_max_ = 0.582	*l* = −15→15
9596 measured reflections	

### Refinement

**Table:**

Refinement on *F*^2^	Primary atom site location: structure-invariant direct methods
Least-squares matrix: full	Secondary atom site location: difference Fourier map
*R*[*F*^2^ > 2σ(*F*^2^)] = 0.041	Hydrogen site location: inferred from neighbouring sites
*wR*(*F*^2^) = 0.118	H atoms treated by a mixture of independent and constrained refinement
*S* = 1.02	*w* = 1/[σ^2^(*F*_o_^2^) + (0.076*P*)^2^] where *P* = (*F*_o_^2^ + 2*F*_c_^2^)/3
1441 reflections	(Δ/σ)_max_ = 0.001
129 parameters	Δρ_max_ = 2.11 e Å^−^^3^
1 restraint	Δρ_min_ = −1.16 e Å^−^^3^

### Special details

**Table:**

Geometry. All e.s.d.'s (except the e.s.d. in the dihedral angle between two l.s. planes) are estimated using the full covariance matrix. The cell e.s.d.'s are taken into account individually in the estimation of e.s.d.'s in distances, angles and torsion angles; correlations between e.s.d.'s in cell parameters are only used when they are defined by crystal symmetry. An approximate (isotropic) treatment of cell e.s.d.'s is used for estimating e.s.d.'s involving l.s. planes.
Refinement. Refinement of *F*^2^ against ALL reflections. The weighted *R*-factor *wR* and goodness of fit *S* are based on *F*^2^, conventional *R*-factors *R* are based on *F*, with *F* set to zero for negative *F*^2^. The threshold expression of *F*^2^ > σ(*F*^2^) is used only for calculating *R*-factors(gt) *etc*. and is not relevant to the choice of reflections for refinement. *R*-factors based on *F*^2^ are statistically about twice as large as those based on *F*, and *R*- factors based on ALL data will be even larger.

### Fractional atomic coordinates and isotropic or equivalent isotropic displacement parameters (Å^2^)

**Table:**

	*x*	*y*	*z*	*U*_iso_*/*U*_eq_	
Ge1	0.12245 (4)	0.12598 (6)	0.00099 (3)	0.0080 (2)	
Ge2	0.37958 (4)	0.11778 (5)	0.25132 (4)	0.0076 (2)	
Cu1	0.25095 (4)	0.37556 (6)	0.12703 (4)	0.0059 (2)	
Na1	0.5000	0.0000	0.5000	0.0192 (8)	
Na2	0.5000	0.4894 (4)	0.2500	0.0183 (8)	
Na3	0.14907 (19)	0.2426 (3)	0.37481 (16)	0.0160 (5)	
O1	0.1236 (3)	−0.0089 (4)	0.0976 (3)	0.0112 (9)	
O2	0.1320 (3)	0.0301 (5)	−0.1209 (3)	0.0133 (8)	
H2	0.0851	0.0580	−0.1585	0.070 (18)*	
O3	0.0009 (3)	0.2188 (5)	0.0010 (3)	0.0137 (9)	
H3	−0.0382	0.1736	−0.0380	0.070 (18)*	
O4	0.2169 (3)	0.2639 (5)	0.0013 (3)	0.0108 (8)	
O5	0.3686 (3)	0.0205 (5)	0.3725 (3)	0.0140 (9)	
H5	0.3402	0.0765	0.4144	0.070 (18)*	
O6	0.5000	0.2115 (6)	0.2500	0.0106 (11)	
O7	0.1210 (3)	0.4809 (4)	0.1556 (3)	0.0121 (8)	
O8	0.2863 (3)	0.2583 (4)	0.2509 (3)	0.0105 (7)	
O1W	0.0000	0.2609 (7)	0.2500	0.0170 (12)	
H1	0.023 (5)	0.333 (6)	0.212 (5)	0.020*	

### Atomic displacement parameters (Å^2^)

**Table:**

	*U*^11^	*U*^22^	*U*^33^	*U*^12^	*U*^13^	*U*^23^
Ge1	0.0107 (4)	0.0072 (4)	0.0060 (3)	−0.00028 (19)	−0.0002 (2)	0.00003 (17)
Ge2	0.0096 (3)	0.0070 (3)	0.0062 (3)	0.00035 (18)	−0.0004 (2)	−0.0002 (2)
Cu1	0.0074 (4)	0.0073 (4)	0.0031 (4)	0.0001 (2)	−0.0005 (2)	−0.0002 (2)
Na1	0.0245 (19)	0.0161 (18)	0.0170 (18)	0.0027 (13)	−0.0063 (16)	−0.0008 (11)
Na2	0.0247 (18)	0.0139 (17)	0.0163 (17)	0.000	0.0081 (16)	0.000
Na3	0.0181 (11)	0.0138 (12)	0.0160 (11)	−0.0008 (8)	−0.0016 (9)	0.0011 (9)
O1	0.012 (2)	0.0102 (18)	0.0114 (19)	0.0010 (13)	0.0034 (16)	0.0044 (14)
O2	0.015 (2)	0.013 (2)	0.0116 (18)	−0.0015 (15)	0.0031 (18)	0.0007 (15)
O3	0.0143 (19)	0.013 (2)	0.013 (2)	0.0034 (15)	0.001 (2)	−0.0005 (13)
O4	0.0148 (19)	0.013 (2)	0.0050 (15)	−0.0053 (16)	−0.0005 (15)	−0.0022 (14)
O5	0.017 (2)	0.012 (2)	0.0123 (19)	0.0009 (15)	0.000 (2)	0.0003 (16)
O6	0.007 (2)	0.011 (3)	0.014 (2)	0.000	−0.002 (2)	0.000
O7	0.012 (2)	0.0115 (19)	0.0130 (19)	−0.0026 (14)	−0.0013 (16)	−0.0037 (15)
O8	0.0126 (18)	0.0107 (18)	0.0081 (15)	0.0021 (14)	0.0001 (15)	0.0047 (16)
O1W	0.023 (3)	0.012 (3)	0.016 (3)	0.000	0.006 (3)	0.000

### Geometric parameters (Å, °)

**Table:**

Ge1—O1	1.721 (4)	Na2—O1W^vi^	2.375 (8)
Ge1—O4	1.724 (4)	Na2—O2^v^	2.408 (4)
Ge1—O3	1.781 (4)	Na2—O2^vii^	2.408 (4)
Ge1—O2	1.795 (4)	Na2—O6	2.429 (6)
Ge2—O7^i^	1.724 (4)	Na2—O1^ii^	2.551 (4)
Ge2—O8	1.729 (3)	Na2—O1^vi^	2.551 (4)
Ge2—O6	1.772 (2)	Na3—O2^viii^	2.395 (5)
Ge2—O5	1.794 (4)	Na3—O4^v^	2.398 (4)
Cu1—O4	1.953 (4)	Na3—O8	2.410 (4)
Cu1—O1^ii^	1.960 (4)	Na3—O5^ii^	2.441 (5)
Cu1—O8	1.961 (4)	Na3—O1W	2.535 (2)
Cu1—O7	1.964 (4)	Na3—O3^ix^	2.543 (5)
Na1—O5^iii^	2.388 (4)	O2—H2	0.8200
Na1—O3^iv^	2.459 (4)	O3—H3	0.8200
Na1—O3^v^	2.459 (4)	O5—H5	0.8200
Na1—O7^iv^	2.568 (4)	O1W—H1	0.85 (4)
Na1—O7^v^	2.568 (4)		
			
O1—Ge1—O4	118.13 (19)	O1W^vi^—Na2—Na3^ii^	48.81 (5)
O1—Ge1—O3	108.67 (19)	O2^v^—Na2—Na3^ii^	45.42 (11)
O4—Ge1—O3	108.5 (2)	O2^vii^—Na2—Na3^ii^	142.64 (15)
O1—Ge1—O2	108.73 (19)	O6—Na2—Na3^ii^	131.19 (5)
O4—Ge1—O2	106.20 (17)	O1^ii^—Na2—Na3^ii^	90.23 (11)
O3—Ge1—O2	105.96 (18)	O1^vi^—Na2—Na3^ii^	89.31 (11)
O7^i^—Ge2—O8	119.18 (18)	Na3^vi^—Na2—Na3^ii^	97.61 (10)
O7^i^—Ge2—O6	108.55 (17)	O2^viii^—Na3—O4^v^	91.63 (15)
O8—Ge2—O6	107.15 (19)	O2^viii^—Na3—O8	98.11 (15)
O7^i^—Ge2—O5	107.56 (19)	O4^v^—Na3—O8	85.22 (14)
O8—Ge2—O5	106.51 (18)	O2^viii^—Na3—O5^ii^	169.22 (15)
O6—Ge2—O5	107.36 (15)	O4^v^—Na3—O5^ii^	95.79 (16)
O7^i^—Ge2—Na3	122.03 (14)	O8—Na3—O5^ii^	90.31 (14)
O6—Ge2—Na3	127.45 (13)	O2^viii^—Na3—O1W	90.37 (19)
O5—Ge2—Na3	71.65 (14)	O4^v^—Na3—O1W	175.92 (17)
O4—Cu1—O1^ii^	106.53 (14)	O8—Na3—O1W	98.03 (12)
O4—Cu1—O8	118.46 (17)	O5^ii^—Na3—O1W	81.77 (18)
O1^ii^—Cu1—O8	103.48 (14)	O2^viii^—Na3—O3^ix^	80.36 (15)
O4—Cu1—O7	101.33 (15)	O4^v^—Na3—O3^ix^	97.19 (14)
O1^ii^—Cu1—O7	120.99 (16)	O8—Na3—O3^ix^	177.16 (16)
O8—Cu1—O7	107.05 (14)	O5^ii^—Na3—O3^ix^	90.94 (15)
O4—Cu1—O7	101.33 (15)	O1W—Na3—O3^ix^	79.63 (12)
O1^ii^—Cu1—O7	120.99 (16)	O2^viii^—Na3—Na2^xi^	45.72 (11)
O8—Cu1—O7	107.05 (14)	O4^v^—Na3—Na2^xi^	137.34 (13)
O7—Cu1—O7	0.0 (2)	O8—Na3—Na2^xi^	98.36 (11)
O5^iii^—Na1—O3^iv^	85.71 (13)	O5^ii^—Na3—Na2^xi^	126.51 (13)
O5^iii^—Na1—O3^v^	94.29 (13)	O1W—Na3—Na2^xi^	44.81 (16)
O3^iv^—Na1—O3^v^	180.0	O3^ix^—Na3—Na2^xi^	78.85 (11)
O5^iii^—Na1—O7^iv^	95.68 (12)	O2^viii^—Na3—Na1^xii^	126.64 (12)
O3^iv^—Na1—O7^iv^	85.86 (13)	O4^v^—Na3—Na1^xii^	96.07 (12)
O3^v^—Na1—O7^iv^	94.14 (13)	O8—Na3—Na1^xii^	135.08 (12)
O5^iii^—Na1—O7^v^	84.32 (12)	O5^ii^—Na3—Na1^xii^	44.80 (10)
O3^iv^—Na1—O7^v^	94.14 (13)	O1W—Na3—Na1^xii^	79.88 (12)
O3^v^—Na1—O7^v^	85.86 (13)	O3^ix^—Na3—Na1^xii^	46.31 (10)
O7^iv^—Na1—O7^v^	180.0	Na2^xi^—Na3—Na1^xii^	109.67 (7)
O5^iii^—Na1—Na3^i^	133.93 (11)	O2^viii^—Na3—Ge2	77.56 (11)
O3^iv^—Na1—Na3^i^	48.40 (10)	O4^v^—Na3—Ge2	71.75 (11)
O3^v^—Na1—Na3^i^	131.60 (10)	O8—Na3—Ge2	25.09 (8)
O7^iv^—Na1—Na3^i^	86.13 (9)	O5^ii^—Na3—Ge2	112.20 (12)
O7^v^—Na1—Na3^i^	93.87 (9)	O1W—Na3—Ge2	112.18 (8)
O5^iii^—Na1—Na3^x^	46.07 (11)	O3^ix^—Na3—Ge2	154.87 (13)
O3^iv^—Na1—Na3^x^	131.60 (10)	Na2^xi^—Na3—Ge2	93.97 (6)
O3^v^—Na1—Na3^x^	48.40 (10)	Na1^xii^—Na3—Ge2	154.09 (7)
O7^iv^—Na1—Na3^x^	93.87 (9)	Ge1—O1—Cu1^i^	120.2 (2)
O7^v^—Na1—Na3^x^	86.13 (9)	Ge1—O1—Na2^xi^	124.3 (2)
Na3^i^—Na1—Na3^x^	180.0	Cu1^i^—O1—Na2^xi^	111.90 (16)
O1W^vi^—Na2—O2^v^	94.04 (13)	Ge1—O2—H2	109.5
O1W^vi^—Na2—O2^vii^	94.04 (13)	Ge1—O3—H3	109.5
O2^v^—Na2—O2^vii^	171.9 (3)	Ge1—O4—Cu1	120.93 (19)
O1W^vi^—Na2—O6	180.000 (1)	Ge1—O4—Na3^xiii^	120.2 (2)
O2^v^—Na2—O6	85.96 (12)	Cu1—O4—Na3^xiii^	114.70 (16)
O2^vii^—Na2—O6	85.96 (12)	Ge2—O5—H5	109.5
O1W^vi^—Na2—O1^ii^	89.65 (11)	Ge2—O6—Ge2^xiv^	124.9 (3)
O2^v^—Na2—O1^ii^	95.04 (12)	Ge2—O6—Na2	117.56 (15)
O2^vii^—Na2—O1^ii^	85.01 (12)	Ge2^xiv^—O6—Na2	117.56 (15)
O6—Na2—O1^ii^	90.35 (12)	Ge2^ii^—O7—Cu1	117.7 (2)
O1W^vi^—Na2—O1^vi^	89.65 (12)	Ge2^ii^—O7—Na1^xiii^	121.2 (2)
O2^v^—Na2—O1^vi^	85.01 (12)	Cu1—O7—Na1^xiii^	114.33 (16)
O2^vii^—Na2—O1^vi^	95.04 (12)	Ge2—O8—Cu1	122.7 (2)
O6—Na2—O1^vi^	90.35 (12)	Ge2—O8—Na3	118.67 (19)
O1^ii^—Na2—O1^vi^	179.3 (2)	Cu1—O8—Na3	113.70 (15)
O1W^vi^—Na2—Na3^vi^	48.81 (5)	Na2^xi^—O1W—Na3^ix^	86.38 (15)
O2^v^—Na2—Na3^vi^	142.64 (15)	Na2^xi^—O1W—Na3	86.38 (15)
O2^vii^—Na2—Na3^vi^	45.42 (11)	Na3^ix^—O1W—Na3	172.8 (3)
O6—Na2—Na3^vi^	131.19 (5)	Na2^xi^—O1W—H1	138 (4)
O1^ii^—Na2—Na3^vi^	89.31 (11)	Na3^ix^—O1W—H1	87 (5)
O1^vi^—Na2—Na3^vi^	90.23 (11)	Na3—O1W—H1	98 (5)
			
O7^i^—Ge2—Na3—O2^viii^	47.40 (19)	O8—Ge2—O6—Na2	0.66 (13)
O8—Ge2—Na3—O2^viii^	144.2 (2)	O5—Ge2—O6—Na2	−113.42 (14)
O6—Ge2—Na3—O2^viii^	−150.40 (15)	Na3—Ge2—O6—Na2	−33.55 (8)
O5—Ge2—Na3—O2^viii^	−52.23 (17)	O2^v^—Na2—O6—Ge2	43.59 (9)
O7^i^—Ge2—Na3—O4^v^	143.33 (18)	O2^vii^—Na2—O6—Ge2	−136.41 (9)
O8—Ge2—Na3—O4^v^	−119.8 (2)	O1^ii^—Na2—O6—Ge2	−51.44 (9)
O6—Ge2—Na3—O4^v^	−54.47 (16)	O1^vi^—Na2—O6—Ge2	128.56 (9)
O5—Ge2—Na3—O4^v^	43.70 (17)	Na3^vi^—Na2—O6—Ge2	−140.83 (6)
O7^i^—Ge2—Na3—O8	−96.8 (3)	Na3^ii^—Na2—O6—Ge2	39.17 (6)
O6—Ge2—Na3—O8	65.4 (2)	O2^v^—Na2—O6—Ge2^xiv^	−136.41 (9)
O5—Ge2—Na3—O8	163.5 (3)	O2^vii^—Na2—O6—Ge2^xiv^	43.59 (9)
O7^i^—Ge2—Na3—O5^ii^	−127.81 (18)	O1^ii^—Na2—O6—Ge2^xiv^	128.56 (9)
O8—Ge2—Na3—O5^ii^	−31.0 (2)	O1^vi^—Na2—O6—Ge2^xiv^	−51.44 (9)
O6—Ge2—Na3—O5^ii^	34.39 (18)	Na3^vi^—Na2—O6—Ge2^xiv^	39.17 (6)
O5—Ge2—Na3—O5^ii^	132.6 (2)	Na3^ii^—Na2—O6—Ge2^xiv^	−140.83 (6)
O7^i^—Ge2—Na3—O1W	−37.9 (2)	O4—Cu1—O7—O7	0.00 (9)
O8—Ge2—Na3—O1W	59.0 (3)	O1^ii^—Cu1—O7—O7	0.00 (16)
O6—Ge2—Na3—O1W	124.35 (18)	O8—Cu1—O7—O7	0.00 (11)
O5—Ge2—Na3—O1W	−137.5 (2)	O4—Cu1—O7—Ge2^ii^	−170.6 (2)
O7^i^—Ge2—Na3—O3^ix^	76.5 (3)	O1^ii^—Cu1—O7—Ge2^ii^	−53.3 (3)
O8—Ge2—Na3—O3^ix^	173.3 (4)	O8—Cu1—O7—Ge2^ii^	64.6 (2)
O6—Ge2—Na3—O3^ix^	−121.3 (3)	O7—Cu1—O7—Ge2^ii^	0(49)
O5—Ge2—Na3—O3^ix^	−23.2 (3)	O4—Cu1—O7—Na1^xiii^	−18.9 (2)
O7^i^—Ge2—Na3—Na2^xi^	4.45 (16)	O1^ii^—Cu1—O7—Na1^xiii^	98.39 (19)
O8—Ge2—Na3—Na2^xi^	101.3 (2)	O8—Cu1—O7—Na1^xiii^	−143.67 (16)
O6—Ge2—Na3—Na2^xi^	166.65 (12)	O7—Cu1—O7—Na1^xiii^	0(22)
O5—Ge2—Na3—Na2^xi^	−95.19 (15)	O7^i^—Ge2—O8—Cu1	−48.1 (3)
O7^i^—Ge2—Na3—Na1^xii^	−151.8 (2)	O6—Ge2—O8—Cu1	75.6 (2)
O8—Ge2—Na3—Na1^xii^	−54.9 (2)	O5—Ge2—O8—Cu1	−169.8 (2)
O6—Ge2—Na3—Na1^xii^	10.4 (2)	Na3—Ge2—O8—Cu1	−153.5 (4)
O5—Ge2—Na3—Na1^xii^	108.6 (2)	O7^i^—Ge2—O8—Na3	105.4 (2)
O4—Ge1—O1—Cu1^i^	−51.6 (3)	O6—Ge2—O8—Na3	−130.96 (16)
O3—Ge1—O1—Cu1^i^	−175.7 (2)	O5—Ge2—O8—Na3	−16.3 (3)
O2—Ge1—O1—Cu1^i^	69.4 (3)	O4—Cu1—O8—Ge2	52.6 (3)
O4—Ge1—O1—Na2^xi^	105.0 (3)	O1^ii^—Cu1—O8—Ge2	−65.0 (3)
O3—Ge1—O1—Na2^xi^	−19.1 (3)	O7—Cu1—O8—Ge2	166.1 (2)
O2—Ge1—O1—Na2^xi^	−134.0 (2)	O7—Cu1—O8—Ge2	166.1 (2)
O1—Ge1—O4—Cu1	−47.6 (3)	O4—Cu1—O8—Na3	−102.1 (2)
O3—Ge1—O4—Cu1	76.6 (3)	O1^ii^—Cu1—O8—Na3	140.33 (17)
O2—Ge1—O4—Cu1	−169.9 (2)	O7—Cu1—O8—Na3	11.5 (2)
O1—Ge1—O4—Na3^xiii^	108.2 (2)	O7—Cu1—O8—Na3	11.5 (2)
O3—Ge1—O4—Na3^xiii^	−127.6 (2)	O2^viii^—Na3—O8—Ge2	−35.2 (2)
O2—Ge1—O4—Na3^xiii^	−14.1 (3)	O4^v^—Na3—O8—Ge2	55.8 (2)
O1^ii^—Cu1—O4—Ge1	170.2 (2)	O5^ii^—Na3—O8—Ge2	151.5 (2)
O8—Cu1—O4—Ge1	54.2 (3)	O1W—Na3—O8—Ge2	−126.7 (2)
O7—Cu1—O4—Ge1	−62.5 (3)	Na2^xi^—Na3—O8—Ge2	−81.4 (2)
O7—Cu1—O4—Ge1	−62.5 (3)	Na1^xii^—Na3—O8—Ge2	149.58 (14)
O1^ii^—Cu1—O4—Na3^xiii^	13.1 (2)	O2^viii^—Na3—O8—Cu1	120.56 (19)
O8—Cu1—O4—Na3^xiii^	−102.9 (2)	O4^v^—Na3—O8—Cu1	−148.5 (2)
O7—Cu1—O4—Na3^xiii^	140.47 (18)	O5^ii^—Na3—O8—Cu1	−52.70 (19)
O7—Cu1—O4—Na3^xiii^	140.47 (18)	O1W—Na3—O8—Cu1	29.0 (2)
O7^i^—Ge2—O6—Ge2^xiv^	−49.42 (14)	Na2^xi^—Na3—O8—Cu1	74.33 (17)
O8—Ge2—O6—Ge2^xiv^	−179.34 (13)	Na1^xii^—Na3—O8—Cu1	−54.7 (3)
O5—Ge2—O6—Ge2^xiv^	66.58 (14)	Ge2—Na3—O8—Cu1	155.8 (3)
Na3—Ge2—O6—Ge2^xiv^	146.45 (8)	O2^viii^—Na3—O1W—Na2^xi^	−4.43 (10)
O7^i^—Ge2—O6—Na2	130.58 (14)		

Symmetry codes: (i) −*x*+1/2, *y*−1/2, *z*; (ii) −*x*+1/2, *y*+1/2, *z*; (iii) −*x*+1, −*y*, −*z*+1; (iv) *x*+1/2, *y*−1/2, −*z*+1/2; (v) −*x*+1/2, −*y*+1/2, *z*+1/2; (vi) *x*+1/2, *y*+1/2, −*z*+1/2; (vii) *x*+1/2, −*y*+1/2, −*z*; (viii) *x*, −*y*, *z*+1/2; (ix) −*x*, *y*, −*z*+1/2;  (x) *x*+1/2, −*y*+1/2, −*z*+1; (xi) *x*−1/2, *y*−1/2, −*z*+1/2; (xii) *x*−1/2, −*y*+1/2, −*z*+1; (xiii) −*x*+1/2, −*y*+1/2, *z*−1/2; (xiv) −*x*+1, *y*, −*z*+1/2.

### Hydrogen-bond geometry (Å, °)

**Table:**

*D*—H···*A*	*D*—H	H···*A*	*D*···*A*	*D*—H···*A*
O2—H2···O6^xiii^	0.82	2.59	3.298 (5)	146
O3—H3···O1^xv^	0.82	1.98	2.765 (6)	161
O5—H5···O4^v^	0.82	1.94	2.755 (6)	171
O1W—H1···O7	0.85 (4)	1.96 (5)	2.773 (6)	159 (6)

Symmetry codes: (xiii) −*x*+1/2, −*y*+1/2, *z*−1/2; (xv) −*x*, −*y*, −*z*; (v) −*x*+1/2, −*y*+1/2, *z*+1/2.
